# Therapeutic sensitivity to Rac GTPase inhibition requires consequential suppression of mTORC1, AKT, and MEK signaling in breast cancer

**DOI:** 10.18632/oncotarget.15586

**Published:** 2017-02-21

**Authors:** Riley A. Hampsch, Kevin Shee, Darcy Bates, Lionel D. Lewis, Laurent Désiré, Bertrand Leblond, Eugene Demidenko, Kurtis Stefan, Yina H. Huang, Todd W. Miller

**Affiliations:** ^1^ Department of Molecular & Systems Biology, Norris Cotton Cancer Center, Geisel School of Medicine at Dartmouth, Lebanon, NH, USA; ^2^ Department of Medicine, Norris Cotton Cancer Center, Geisel School of Medicine at Dartmouth, Lebanon, NH, USA; ^3^ Department of Community & Family Medicine, Norris Cotton Cancer Center, Geisel School of Medicine at Dartmouth, Lebanon, NH, USA; ^4^ Diaxonhit, Paris, France; ^5^ Department of Microbiology and Immunology, Norris Cotton Cancer Center, Geisel School of Medicine at Dartmouth, Lebanon, NH, USA; ^6^ Comprehensive Breast Program, Norris Cotton Cancer Center, Geisel School of Medicine at Dartmouth, Lebanon, NH, USA

**Keywords:** Rac1, Rac3, ERK, mTOR, breast cancer

## Abstract

Rac GTPases have oncogenic roles in cell growth, survival, and migration. We tested response to the Rac inhibitor EHT1864 in a panel of breast cancer cell lines. EHT1864-induced growth inhibition was associated with dual inhibition of the PI3K/AKT/mTORC1 and MEK/ERK pathways. Breast cancer cells harboring *PIK3CA* mutations or HER2 overexpression were most sensitive to Rac inhibition, suggesting that such oncogenic alterations link Rac activation with PI3K/AKT/mTORC1 and MEK/ERK signaling. Interestingly, EHT1864 decreased activation of the mTORC1 substrate p70S6K earlier than AKT inhibition, suggesting that Rac may activate mTORC1/p70S6K independently of AKT. Comparison of the growth-inhibitory profile of EHT1864 to 137 other anti-cancer drugs across 656 cancer cell lines revealed significant correlation with the p70S6K inhibitor PF-4708671. We confirmed that Rac complexes contain MEK1/2 and ERK1/2, but also contain p70S6K; these interactions were disrupted by EHT1864. Pharmacokinetic profiles revealed that EHT1864 was present in mouse plasma at concentrations effective *in vitro* for approximately 1 h after intraperitoneal administration. EHT1864 suppressed growth of HER2+ tumors, and enhanced response to anti-estrogen treatment in ER+ tumors. Further therapeutic development of Rac inhibitors for HER2+ and *PIK3CA*-mutant cancers is warranted.

## INTRODUCTION

Rac GTPases (Rac1/1b/2/3) have been implicated in cancer cell motility, survival, and proliferation. The three highly homologous Rac proteins are encoded by separate genes (*RAC1/2/3*), and isoform-specific functions remain to be fully elucidated. Rac1 contains a distinct carboxyl terminus that drives oligomerization and possibly nuclear translocation [1, 2]. Rac1b is a constitutively active splice variant ot Rac1 overexpressed in breast and other cancers [3]. Rac2 is primarily expressed in hematopoetic cells [4]. Rac3 is primarily expressed in brain tissues, and has been shown to be dysregulated in ovarian, breast, gastric, and brain cancers [5, 6]. While activating mutations in *RAC1/2/3* are rare, Rac hyperactivation is a common theme in many cancers including breast cancer [7–12]. Aberrant Rac signaling frequently occurs through Rac guanine exchange factor hyperactivation resulting from deregulated upstream signaling events. Rac-activating GEFs such as Tiam1, Trio, Vav3, and PREX-1 are overexpressed in breast tumors [8–11]. Canonical Rac signaling involves activation of p21-activated kinases (PAKs), which in turn activate mitogen-activated protein kinases (MEK1/2 and ERK1/2) to drive proliferation and survival pathways [13]. Mounting evidence suggests that Rac plays a key role in the phosphatidylinositol 3-kinase (PI3K) pathway [14–16]. Class IA PI3Ks are typically activated by receptor tyrosine kinases and G protein-coupled receptors. PI3K phosphorylates phosphatidylinositol (4,5)-bisphosphate (PIP_2_) to create the 3,4,5-trisphosphate (PIP_3_) at the plasma membrane, and PIP_3_ recruits intracellular pleckstrin homology (PH) domain-containing proteins such as AKT for activation. Rac1 directly binds and activates the p110β isoform of PI3K [14]. We recently described a positive feedback loop where Rac signaling drives activation of receptor tyrosine kinase (RTK)/PI3K pathways that activate PREX-1 in breast cancer [15].

The PI3K/AKT/mechanistic target of rapamycin (mTOR) pathway promotes cell growth, proliferation, migration, and survival, and as such, aberrations within this signaling axis occur in the majority of breast and other cancers [17]. Several inhibitors of PI3K and mTOR are in clinical trials for estrogen receptor α-positive (ER+) and HER2-overexpressing (HER2+) breast cancers. mTOR exists in two complexes, mTORC1 and mTORC2, that lie upstream and downstream of AKT, respectively [18, 19]. The mTORC1 inhibitor everolimus is approved for the treatment of advanced ER+ breast cancer. While these drugs have shown encouraging clinical results, efficacy may be limited due to extensive cross-talk and compensatory feedback upregulation of MEK/ERK and RTK signaling, and upregulation of PI3K/AKT signaling by mTORC1 inhibition [20–23]. Preclinical studies testing combinations of PI3K/AKT/mTOR and MEK/ERK pathway-directed inhibitors have shown impressive anti-tumor effects in a variety of cancer subtypes, but these drug combinations have proven toxic in humans [24, 25]. With evidence implicating Rac in both of these key oncogenic signaling pathways, we investigated the therapeutic potential of inhibiting Rac activity as a means to simultaneously target the PI3K and MEK pathways in breast cancer.

## RESULTS

### Rac inhibition suppresses growth and induces apoptosis in breast cancer cells

**Figure 1:**
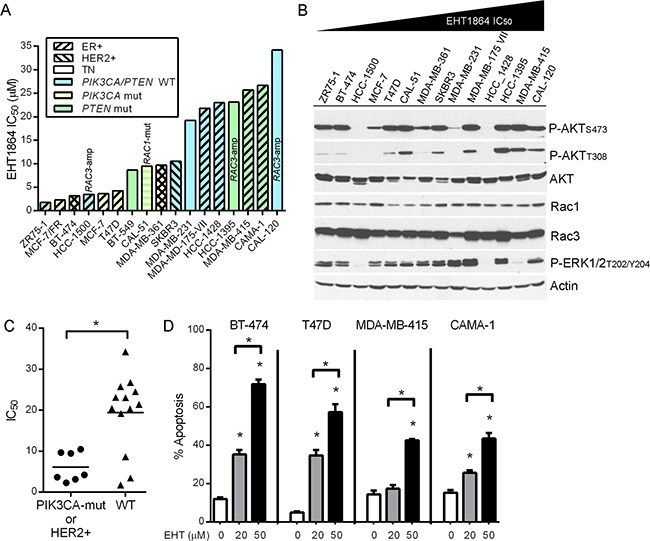
Rac inhibition suppresses growth and induces apoptosis in *PIK3CA*-mutant and HER2+ breast cancer cells **(A)** Breast cancer cells were treated with 0-100 μM EHT1864 for 4-5 d. Relative viable cell numbers were assessed by SRB assay. Mutational and DNA copy number profiles were obtained from ref. [53]. *RAC3*-amp-*RAC3* gene amplification. *RAC1*-mut- *RAC1*^N39S^ mutation, predicted to be low-impact permutationassessor.org [54]. MCF-7/FR- fulvestrant-resistant MCF-7 cells maintained and treated in 1 μM fulv. **(B)** Cell lysates were analyzed by immunoblot. **(C)** Comparison of IC_50_ values between cells harboring a *PIK3CA* mutation and/or HER2 amplification vs. *PIK3CA*/HER2-wild-type cells. *p=0.015 by Mann-Whitney U-test. **(D)** Cells were treated with EHT for 72 h before apoptosis assay. *p<0.0001 by Bonferonni post-hoc test compared to control for each cell line unless otherwise indicated with brackets.

The small molecule EHT1864 binds Rac1/1b/2/3 and promotes loss of guanine nucleotide association, locking Rac in an inactive conformation, and inhibiting GTPase activity and engagement of downstream effectors. EHT1864 blocks activation of Rac, but not the related proteins CDC42 or RhoA, at a concentration of 50 μM in glioblastoma cells [26, 27]. We screened 17 human breast cancer cell lines for sensitivity to EHT1864 in growth assays. IC_50_ values ranged from 2.0 to 39.1 μM (Figure 1A and Supplementary Figure 1). Relative levels of activation of the PI3K/AKT pathway [assessed by phospho-AKT_T308_ and phospho-AKT_S473_ as respective markers of phosphatidylinositol 3,4,5-trisphosphate (PIP_3_) levels and mTORC2 activity] and the MEK/ERK pathway (assessed by P-ERK1/2), or levels of Rac1 and Rac3 did not generally correlate with sensitivity to EHT1864 (Figure 1B). Three tested cell lines harbor *RAC3* genomic amplification, but this aberration also did not correlate with EHT1864 sensitivity. Interestingly, cell lines that harbor activating mutations in the gene encoding the p110α catalytic subunit of PI3K (*PIK3CA*), or amplification of the *ERBB2* (HER2) proto-oncogene showed significantly increased sensitivity to EHT1864 (Figure 1C). EHT1864 also induced apoptosis in 4/4 breast cancer cell lines tested in a dose-dependent manner. Notably, Rac inhibition induced a greater degree of apoptosis (compared to baseline) in BT-474 and T47D cells, which had lower IC_50_ values in growth assays, compared to MDA-MB-415 and CAMA-1 cells (Figure 1D).

### Sensitivity to Rac inhibition is associated with dual inhibition of MEK/ERK and PI3K/AKT/mTOR pathways

**Figure 2:**
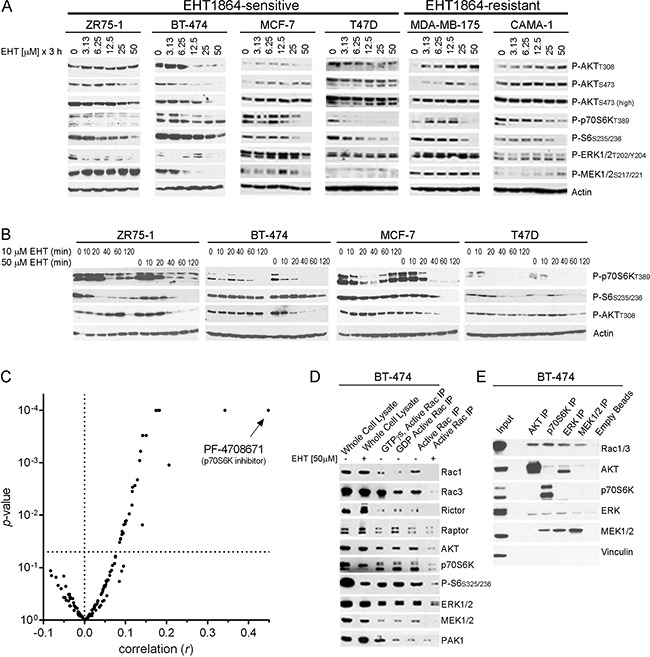
Sensitivity to Rac inhibition is associated with dual inhibition of MEK/ERK and PI3K/AKT/mTORC1 pathways **(A–B)** Cells were treated with 0-50 μM EHT1864 for 3 h (A), or 10 or 50 μM EHT1864 for 0-120 min (B), and lysates were analyzed by immunoblot. Cells that are relatively sensitive vs. resistant to EHT1864 (from Figure 1A) are indicated. **(C)** Mining of sensitivity data from 656 cancer cell lines treated with a panel of 138 drugs [28] revealed that the sensitivity profile of EHT1864 most strongly correlates with the profile of the p70S6K inhibitor PF-4708671. **(D–E)** In (D), activated Rac was pulled down under control-, GTPγs-, GDP-, and EHT1864-treated conditions. In (E), AKT, p70S6K, ERK, and MEK1/2 were immunoprecipitated from cell lysates. Eluates and lysates were analyzed by immunoblot.

We previously reported that Rac inhibition suppresses both the MEK/ERK and PI3K/AKT/mTOR pathways in ER+ breast cancer cells [15]. To identify potential differences in Rac signaling between EHT1864-sensitive vs. -resistant breast cancer cells (Figure 1A), we evaluated effects on these oncogenic pathways. In EHT1864-sensitive cells, but not cells with relative drug resistance, EHT1864 treatment decreased levels of both phospho-AKT_T308_ and P-ERK1/2 (Figure 2A), suggesting that dual inhibition of the PI3K/AKT and MEK/ERK pathways is required for sensitivity to Rac inhibition.

In *PIK3CA*-mutant and HER2+ breast cancer cells, PI3K/AKT signaling frequently drives mTORC1, which in turn activates p70S6 kinase (p70S6K) (Supplementary Figure 2). We observed that EHT1864 treatment often induced decreases in phospho-p70S6K at lower doses and earlier time points than decreases in phospho-AKT (Figure 2A/2B). This suggests that EHT1864 inhibits mTORC1/p70S6K activation at a node downstream and independent of AKT. Capitalizing on the public availability of drug sensitivity profiles from 656 cancer cell lines to 138 anti-cancer drugs [28], we compared patterns of IC_50_ values for EHT1864 to each other drug. This analysis revealed that the sensitivity profile of EHT1864 is most strongly correlated with the profile of the p70S6K inhibitor PF-4708671 (Figure 2C), further supporting the notion that the growth-inhibitory effects of Rac inhibition involve p70S6K inhibition.

Active Rac1 has been shown to bind and activate PI3K/p110β [14]. Active Rac1 has been found in complex with mTORC1 and mTORC2, and is thought to direct complex localization [29]. GTP-bound Rac1 has also been shown to bind p70S6K and promote p70S6K activation [30]. To determine whether Rac interacts with proteins in the AKT/mTOR and MEK/ERK signaling cascades in breast cancer cells, we performed Rac pulldown assays: beads coated with protein encoding the p21-binding domain (PBD) of the Rac/CDC42 effector PAK1 are used to capture active GTP-bound Rac and CDC42, which are then detected by immunoblot analysis of bead eluates. Treatment of cell lysates with the non-hydrolyzable nucleotide GTPγs locks Rac in an active conformation, while treatment with GDP stoichiometrically promotes Rac inactivation. In GTPγs-treated lysates of BT-474 cells, increased amounts of Rac1, Rac3, and PAK1 were pulled-down, confirming assay functionality. In untreated lysates, PBD beads pulled-down AKT, the mTORC2 component Rictor, AKT, p70S6K, MEK1/2, and ERK1/2 in a Rac activation-dependent manner, as confirmed by treatment with EHT1864 (Figure 2D). PBD beads also pulled-down Raptor independent of Rac activation. Whether these proteins exist in distinct or overlapping complexes with Rac/CDC42 will require further in-depth study. Reverse immunoprecipitations of AKT, p70S6K, ERK1/2, and MEK1/2 confirmed that these proteins complex with Rac1 and/or Rac3 (Figure 2E). These data also suggested that some proteins exist in unexpected complexes, such as MEK and p70S6K. These observations imply that Rac directly engages components of the PI3K/AKT /mTOR and MEK/ERK axes.

### Constitutive AKT activation does not confer resistance to Rac inhibition

**Figure 3:**
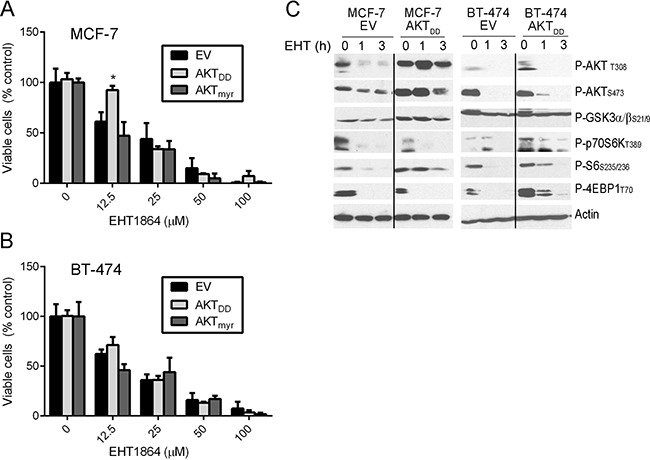
Overexpression of p70S6K confer resistance to EHT1864 **(A–B)** MCF-7 and BT-474 cells were stably transfected with vectors encoding AKT1_DD_ or AKT1_myr_ constitutively active mutants or EV, and sensitivity to EHT1864 was assessed via growth assay. In **(C)** lysates were analyzed by immunoblot. *p<0.05 by Bonferroni multiple comparison-adjusted post-hoc test compared to EV control at each dose of EHT.

Since Rac was found in complex with AKT and p70S6K, and EHT1864 inhibited phosphorylation of p70S6K prior to AKT (Figure 2B/2D/2E), we tested whether AKT inhibition was critical for the growth-suppressive effect of EHT1864. We generated MCF-7 cells stably overexpressing constitutively active AKT (AKT_myr_ or AKT_DD_). AKT activation did not alter sensitivity to EHT1864 (Figure 3A/3B and supplementary Figure 3). EHT1864 treatment decreased phosphorylation of p70S6K and the mTORC1 substrate 4EBP1 despite constitutive AKT activation (assessed by phosphorylation of the AKT substrates GSKα/β) (Figure 3C). These data collectively support a model in which Rac activates mTORC1/p70S6K independent of PI3K/AKT.

### Duration and magnitude of Rac inhibition affect breast cancer cell growth

**Figure 4:**
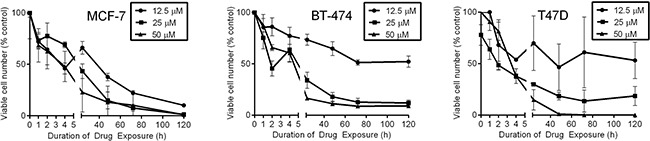
Short-term exposure to EHT1864 elicits prolonged growth-suppressive effects Cells were treated with 12.5, 25, or 50 μM EHT1864 for 0, 1, 2, 4, 8, 24, 48, 72, or 120 hours, then drug was washed out. Relative viable cell numbers were quantified at the 120-h time point.

We and others have shown that transient interruption of oncogenic kinase signaling is sufficient to elicit robust, delayed anti-cancer effects [31–34]. To determine the duration of Rac inhibition required to induce anti-cancer effects in EHT1864-sensitive cells (from Figure 1A), cells were treated with 0-50 μM EHT1864 for 0-120 h, followed by drug washout. Relative numbers of viable cells were measured after 120 h. A 2- to 4-h exposure to 50 μM EHT1864 decreased cell viability ≥50% (Figure 4). In contrast, a lower concentration of EHT1864 (12.5 μM) required longer durations of exposure (48-60 h) to appreciably decrease viability, reflecting a relationship between duration and magnitude of Rac inhibition, and cell viability.

### Pharmacokinetic analysis of EHT1864 in mice

#### Pharmacokinetics of EHT1864 in mice

**Figure 5:**
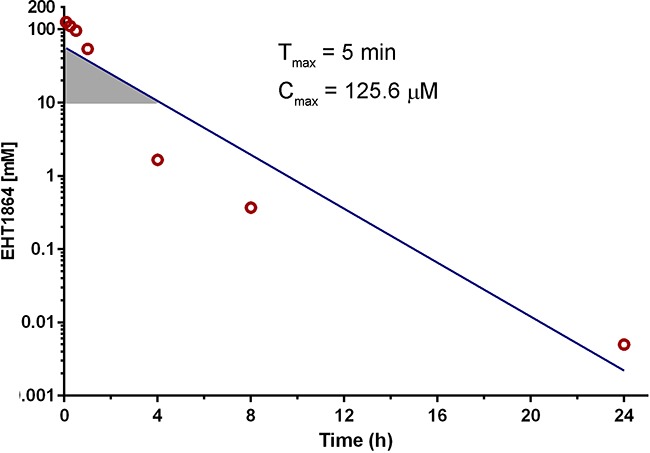
Pharmacokinetic analysis of EHT1864 in mouse plasma Mice were injected i.p. with a single dose of EHT1864 (100 mg/kg), and blood was collected from 3 mice per time point over the next 24 h. Plasma was separated for EHT1864 concentration measurement. The Tmax- time to maximum concentration; Cmax- maximum concentration; were the observed mean values and the terminal elimination half-life was estimated using non compartmental analysis. (mean elimination t_1/2_ = 99.2 min /1.65h). The shaded region indicates estimated time that plasma EHT1864 concentration exceeded 10 μM.

Despite being used as a tool compound in many preclinical studies, the pharmacokinetic properties and anti-tumor efficacy of EHT1864 have not been previously reported. The plasma EHT concentration vs time profile following a single i.p. injection of EHT1864 (100 mg/kg) is shown in Figure 5A. Non-compartmental analysis of the mean plasma EHT1864 concentration versus time data revealed an estimated elimination half-life of 99.2 min (1.65 h).The mean maximum plasma concentration (Cmax) was 125.6 μM (range 107.7-147.8 μM) and the mean Tmax was 5 minutes post injection. After 1 h, the mean plasma concentration of EHT1864 had decreased to 54.1 μM (range 49.1-60 μM), and declined within 4 h to concentrations unlikely to appreciably inhibit cancer cell growth (Figure 5A). Assuming log-linear clearance from plasma, EHT1864 was estimated to be present at ≥10 μM for 1.24 h.

Treatment with 100 mg/kg EHT1864 twice daily was well-tolerated, while 150 mg/kg twice daily caused signs of toxicity (*i.e*., lethargy).

### Rac inhibition suppresses breast tumor growth

**Figure 6:**
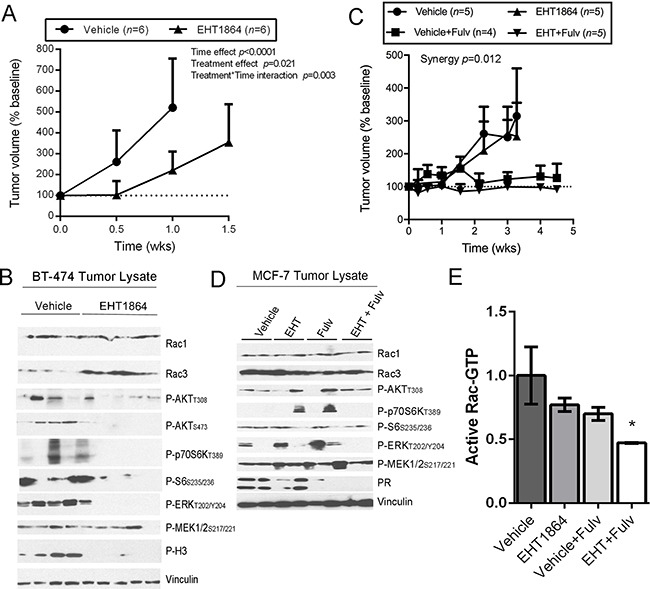
EHT1864 inhibits breast tumor growth **(A, C)** Mice bearing BT-474 tumors (A) or MCF-7 tumors (C) were randomized to drug treatments as indicated. Data are presented as % tumor volume relative to baseline (mean + SEM). **(B, D, E)** After 6 wk of treatment, tumors were harvested at 1 h after the final dose of EHT1864. Lysates were analyzed by immunoblot (B/D), or used for active Rac ELISA (E).

Mice bearing s.c. ER+/HER2+/*PIK3CA*-mutant BT-474 xenografts were treated with EHT1864 (100 mg/kg) or vehicle twice daily. EHT1864 significantly slowed tumor growth compared to vehicle control (Figure 6A and Supplementary Figure 4; mean weekly growth rates of 50% vs. 27%). In tumor specimens acquired after 2 wk of treatment, EHT1864 greatly reduced levels of P-ERK1/2, P-AKT, P-p70S6K, and P-Histone H3_S10_ (marker of mitosis) (Figure 6B).

In contrast, single-agent EHT1864 modestly slowed growth of s.c. ER+/HER2-/*PIK3CA*-mutant MCF-7 xenografts (Figure 6C and Supplementary Figure 5; mean weekly growth rates of 4.32% vs. 3.4%; *p*=0.035). MCF-7 cells require exogenous estrogen supplementation to form tumors in mice [35]. Estrogen-activated ER is a major driver of MCF-7 tumor growth, and inhibition of oncogenic signaling pathways (*e.g*., PI3K/AKT) induces upregulation of ER levels and activation. While single-agent therapies targeting oncogenic pathways are often only modestly effective against ER+ breast tumors, combination treatment with anti-estrogens is frequently more effective than anti-estrogens alone [36, 37]. Indeed, treatment with the anti-estrogen fulvestrant significantly inhibited growth of MCF-7 tumors (*p*<0.0001; mean weekly growth rate of 1.43%), while the combination of EHT1864 and fulvestrant was significantly more effective than either single agent, and provided stable disease (Figure 6C; *p*<0.0001 vs. vehicle; synergy *p*=0.012; mean weekly growth rate of 0.97%). In tumor specimens acquired after 3-6 wk of treatment, we observed that fulvestrant downregulated levels of progesterone receptor (PR), which is encoded by an ER-inducible gene (Figure 6D). EHT1864 reduced the levels of active Rac-GTP (Figure 6E and Supplementary Figure 6), but did not appreciably affect ERK, AKT, or p70S6K phosphorylation (Figure 6D); it is possible that tumor cells adapted to Rac inhibition during treatment to maintain oncogenic pathway activation, and/or the time point selected for analysis was outside the window of pathway inhibition. However, EHT1864 in combination with fulvestrant reduced levels of active Rac, MEK1/2, ERK1/2, and AKT (Figure 6D/6E).

## DISCUSSION

The role of Rac GTPases in cancer processes has been widely documented. However, Rac has remained an elusive therapeutic target. We demonstrate that, among breast cancer cells, mutations in *PIK3CA* and/or HER2 are predictive of increased sensitivity to Rac inhibition with EHT1864. Sensitivity to Rac inhibition was associated with EHT1864-induced decreases in activation of both the AKT/mTOR/p70S6K and MEK/ERK pathways, identifying Rac as a key upstream signaling node in both pathways in Rac-dependent cells. Temporal and dose-response analyses revealed that Rac activates mTORC1/p70S6K independently of AKT; when considered in the context of prior findings [14, 15, 29, 30], these observations place Rac both upstream and downstream of AKT/mTORC1. Despite only providing transient Rac inhibition *in vivo*, EHT1864 significantly inhibited growth of breast tumors in mice.

The PI3K/AKT/mTOR and MEK/ERK pathways are two of the most commonly aberrantly activated pathways in human cancers. Crosstalk and compensatory signaling between these pathways have been widely reported. Although combined therapeutic targeting of these pathways showed impressive preclinical results, such drug combinations elicit considerable toxicity in humans [24, 25], likely because these pathways are essential in many normal cell types. Rac proteins have been directly or indirectly implicated in activation of PI3K/p110β, AKT, mTORC1, mTORC2, p70S6K, MEK1/2, and ERK1/2 [14, 29, 30]. Thus, we considered whether Rac could serve as a single therapeutic target critical to both the PI3K/AKT/mTOR and MEK/ERK pathways. Indeed, Rac inhibition with EHT1864 suppressed growth and induced apoptosis (Figure 1A/1D and Supplementary Figure 1) in cells in which Rac drives both the AKT/mTOR and MEK/ERK pathways (Figure 2A). Among breast cancer cell lines, these features were significantly associated with mutations in *PIK3CA* or amplification of HER2 (Figure 1C), both of which hyperactivate PI3K. Thus, Rac may drive both the AKT/mTOR and MEK/ERK pathways only in select subtypes of cancer cells, making Rac a promising therapeutic target that could supplant that need for drug combinations targeting individual components of both the PI3K/AKT/mTOR and MEK/ERK pathways. Whether Rac drives activation of the PI3K/AKT/mTOR and MEK/ERK pathways by increasing kinase activity and/or decreasing phosphatase activity requires further study.

A prior study by Katz et al. showed that EHT1864 suppressed cancer cell invasion, proliferation, and survival in a three-dimensional triple-negative breast cancer cell line model, and in patient-derived breast tumor tissue organotypic cultures regardless of ER/HER2 status [38]. EHT1864 decreased levels of STAT3 Ser727 (activating) phosphorylation, survivin, and cyclin D1; the latter two proteins are encoded by STAT3 target genes. These effects were recapitulated by treatment with the STAT3 inhibition Stattic, leading the authors to conclude that EHT1864 effects occurred via STAT3 inhibition. The implication of STAT3, cyclin D1, and survivin in response to Rac inhibition does not necessarily conflict with our findings attributing response to inhibition of the PI3K/AKT/mTOR and MEK/ERK pathways. Cyclin D1 translation is mTORC1-dependent [39]. STAT3 can be phosphorylated at Ser727 by mTORC1 or ERK1/2 [40, 41]. Thus, phospho-STAT3_S727_ may be a downstream read-out of the PI3K/AKT/mTOR and MEK/ERK pathways.

In addition to EHT1864, other small molecule inhibitors of Rac are in preclinical development. NSC23766 inhibits interactions between of Rac and GEFs, including Trio and Tiam1, inhibiting Rac activation and cancer cell invasion, metastasis, and neoangiogenesis in multiple cancer subtypes [42]. However, concentrations of NSC23766 required to reach efficacious doses limit its therapeutic potential, and NSC23766 does not inhibit the constitutively active Rac1 splice variant Rac1b. EHop-016, a NSC23766 structure-based Rac inhibitor effective at therapeutically achievable doses, slows tumor metastasis and angiogenesis in breast cancer cell lines by blocking Rac interaction with the GEF Vav. However, EHop-016 allowed Rac interaction with Tiam1 and other GEFs, which may ultimately limit its therapeutic utility [43, 44]. In contrast, EHT1864 is a small molecule pan-Rac inhibitor that locks Rac into an inactive conformation by guanine nucleotide displacement rather than inhibition of Rac-GEF interaction. There have been over 70 Rac GEFs reported, many of which are linked to cancer processes. Molecules such as EHT1864 that inhibit Rac in a GEF-independent manner may be a more promising strategy than targeting GEFs [26, 45].

In summary, these results collectively demonstrate that therapeutic targeting of Rac is a promising therapeutic strategy for breast cancer, particularly in cancers harboring activating mutations in *PIK3CA* or amplification of HER2. Pulsatile treatment studies, combined with pharmacokinetic and tumor growth studies in mice, suggest that transient Rac inhibition elicits significant growth-inhibitory effects. These results warrant further development of Rac inhibitors to improve pharmacologic properties prior to clinical testing.

## MATERIALS AND METHODS

### Cell Culture

CAL-51 and CAL-120 cells were obtained from DSMZ (Braunschweig, Germany). Other parental cell lines were obtained from ATCC. All cell lines were cultured in DMEM with 10% FBS (Hyclone). Fulvestrant-resistant MCF-7/FR cells were obtained from Matthew Ellis (Washington Univ., St. Louis, MO) and maintained in 1 μM fulvestrant (Tocris Biosciences). EHT1864 [27] was generously provided by Diaxonhit (Paris, France). Cells were stably transfected with viral vectors as described in Supplemental Methods.

### Immunoblotting

Immunoblotting of protein extracts from cells and frozen tumor fragments was performed as previously described [46].

### Sulforhodamine B (SRB) growth assay

Cells were plated at 5,000/well in 96-well plates and treated in triplicate as indicated. Relative numbers of adherent cells were determined by SRB staining as previously described [47].

### Apoptosis assay

Cells were plated at 50,000/well in 12-well plates and treated with EHT1864 for 72 h. Twelve hours before analysis, positive and negative control wells were treated with or without 5 μM BKM120 (PI3K inhibitor, Selleck Chemicals), respectively. Floating and adherent cells (dislodged by trypsinization) were processed using ApoScreen Annexin Apoptosis kit (Southern Biotech) and analyzed by flow cytometry. Cells staining positively for Annexin-V were considered apoptotic.

### Active Rac assay

Measurement of active Rac in cell lysates was performed using Rac1 Pull-down Activation Assay Biochem Kit (Cytoskeleton). Cells treated +/− 50 μM EHT1864 for 2 h were lysed on ice in lysis buffer, adjusted for equal protein content (determined by BCA assay, Pierce), and incubated with 10 μL of GST-tagged PAK-PBD beads for 1 h at 4°C per manufacturer's instructions. Positive and negative control lysates were incubated with GTPγS (non-hydrolysable GTP) and GDP, respectively, for 15 min at room temperature prior to incubation with beads. Beads were then washed, and protein was eluted with 1x NuPAGE LDS Sample Buffer (Life Technologies) with 5% β-mercaptoethanol. Eluates and whole-cell lysates were analyzed by immunoblotting.

Measurement of active Rac in tumor lysates was performed using the colorimetric Rac1 G-LISA Activation Assay Kit (Cytoskeleton). Frozen tumor fragments were homogenized in lysis buffer, and protein content was quantified by BCA assay. Active Rac in lysates was bound to wells during a 30-min incubation at 4°C. Recombinant active Rac protein and lysis buffer were used as positive and negative controls, respectively. Wells were then washed and serially incubated with antigen-presenting buffer, anti-Rac1 primary antibody, HRP-conjugated secondary antibody, and HRP-detection reagents per manufacturer's instructions. Relative amounts of active Rac were determined through spectrophotometric readings at 490 nm.

### Pharmacokinetic analyses

All animal studies were approved by the Dartmouth College IACUC. Female NOD-scid/IL2Rγ^−/−^ (NSG; NOD.Cg-Prkdcscid Il2rgtm1Wjl/SzJ) mice (6-7 wks old; obtained from the Norris Cotton Cancer Center Transgenic & Genetic Construct Shared Resource) were treated with 100 mg/kg EHT1864 i.p. Blood was collected by cardiac puncture from 3 mice per time point for up to-24 h post i.p injection. Plasma was separated and stored at −80°c until analyzed. Plasma EHT1864 concentrations were measured using a liquid chromatography (LC) tandem mass spectrometry (MS/MS) assay with technical triplicates (detailed in Suppl. Methods). The pharmacokinetic data analysis (modeling) was undertaken using non compartmental modeling (model 200) in WinNonLn (Pharsight, Mountain View, CA)

### Xenograft studies

Female NSG mice were s.c. injected with 5-10×10^6^ BT-474 or MCF-7 cells. Mice injected with MCF-7 cells were s.c. implanted on the same day with a 17β-estradiol (1 mg) beeswax pellet [48]. When average tumor volume reached 200 mm^3^, mice were randomized to treatment with vehicle, EHT1864 (100 mg/kg, i.p. BID), fulvestrant (5 mg/wk, s.c; clinical formulation kindly provided by AstraZeneca), or the combination. Tumor volumes were measured twice weekly using calipers (volume = length^2^ x width/2). Tumors were harvested for snap-freezing.

### Statistical analyses

*In vitro* cell growth and apoptosis data were analyzed by ANOVA followed by Bonferroni multiple comparison-adjusted post-hoc test between groups. To estimate progression/regression of tumors, the following linear mixed model was employed: log_10_(tumor volume_it_) = a_i_ + b*t + e_it_, where i represents the i-th mouse and t represents the time of tumor volume measurement, a_i_ represents the mouse-specific log tumor volume at the baseline (t=0), slope b represents the rate of tumor volume growth (or reduction), and e_it_ represents the deviation of measurements from the model over time (refs. [49–51]). The variance of a_i_ is interpreted as mouse heterogeneity and b*log_e_(10)*100 estimates the percent tumor volume increase per week. The computation was carried out in statistical package R [52], using function ‘lme’ from library ‘nlme.’ Treatment groups were compared using Z-test for slopes with standard error derived from lme. Synergy (Figure 6) was assessed using the difference of slopes (b_1_-b_0_)+(b_2_-b_0_) - (b_12_-b_0_) where b_1_, b_2_, b_12_, and b_0_ are the slopes from the treatment groups 1 and 2, the combined treatment, and control group.

## SUPPLEMENTARY MATERIALS AND MUTHODS AND FIGURES


