# Supplementary material for: Therapeutic sensitivity to Rac GTPase inhibition requires consequential suppression of mTORC1, AKT, and MEK signaling in breast cancer
Source: Oncotarget. 2017 Feb 21;8(13):21806–17. doi: 10.18632/oncotarget.15586 (PMC5400625; doi:10.18632/oncotarget.15586)
Supplement: Supplementary file 1 [file oncotarget-08-21806-s001.pdf]

Therapeutic sensitivity to Rac GTPase inhibition requires consequential suppression of mTORC1, AKT, and MEK signaling in breast cancer

SUPPLEMENTARY METHODS

Stably transfected cell lines

AKT_{DD}, AKT_{myr}, and vector control stable cell lines were generated using the following retroviral plasmids: pBMN-AKT1DD-HA-I-GFP, pBMN-myrAKT1-HA-I-GFP [1], and pBMN-I-GFP (Addgene). PhoenixGP cells (ATCC) were used to generate retrovirus using standard protocols. Stably transfected target cancer cells were selected for GFP expression by FACS.

Immunoblotting

Cells were lysed, and frozen tumors were homogenized and lysed in RIPA buffer [50 mM Tris pH 7.4, 150 mM NaCl, 1% NP-40, 0.5% deoxycholic acid, 0.1% SDS plus Halt protease inhibitor cocktail (Pierce) and 1 mM Na_3VO_4 (New England Biolabs)]. Lysates were sonicated for 15 sec, centrifuged at 17,000 x *g* for 10 min at 4°C, and protein in supernatants was quantified using BCA assay (Pierce). Protein extracts were denatured with NuPage (Life Technologies), and reduced with 1.25% β-mercaptoethanol (Sigma). Proteins were separated by SDS-PAGE, and transferred to nitrocellulose. Blots were probed with antibodies against P-AKT_{S473}, P-AKT_{T308}, AKT, P-$S6_{S240/244}$, P-p70$S6K_{T389}$, p70S6K, P-ERK$1/2_{T202/Y204}$, ERK1/2, P-MEK$1/2_{S217/221}$, MEK1/2, P-4EB$P1_{T37/46}$, P-PAK$1/2_{T423/402}$, , P-Histone $H3_{S10}$, Rictor, Actin (Cell Signaling), Rac1 (Cytoskeleton), mTOR (Santa Cruz Biotechnology), Raptor, and Rac3 (Abcam). HRP-labeled secondary antibodies (GE Healthcare) and ECL substrate (Pierce) were used for signal detection.

Immunoprecipitation

Cells were lysed in 1% NP-40 buffer [20 mM Tris, pH 7.4, 150 mM NaCl, 1% NP-40, 10% glycerol, 1 mM EDTA, 1 mM EGTA, 5 mM sodium pyrophosphate, 50 mM NaF, 10 mM sodium β-glycerophosphate, 1 mM Na_3VO_4 (New England Biolabs), plus Halt protease inhibitor cocktail (Pierce)]. Lysates were sonicated and cleared by centrifugation at 18000 x *g* for 10 min. Fifteen µL of Dynabeads Protein G slurry (Invitrogen) was incubated with 3 µg of antibody (targeting AKT, p70S6K, ERK1/2, or MEK1/2) and cell lysate overnight at 4°C. Beads were washed with lysis buffer, and protein was eluted with 1x NuPAGE LDS Sample Buffer (Life Technologies) with 5% β-mercaptoethanol. Eluates and whole-cell lysates were analyzed by immunoblotting.

Pharmacokinetic sample collection; EHT1864 plasma concentration measurement

Female NOD-scid/IL2R$\gamma^{-/-}$ (NSG; NOD.Cg-Prkdcscid Il2rgtm1Wjl/SzJ) mice (6-7 wks old) were treated with 100 mg/kg EHT1864 dissolved in water in a 200-µL i.p. injection. Blood was collected by cardiac puncture from 3 mice per time point for up to 24h post injection into tubes containing EDTA (0.5 M final concentration) as an anti-coagulant, and centrifuged at 2,000 x *g* for 5 min at 4°C. Plasma was separated and stored at -80°C until analyzed.

Plasma concentrations of EHT1864 were measured by liquid chromatography (LC) with tandem mass spectrometry (MS/MS) in technical triplicates, and the average value from the technical triplicates for each biological sample was used for calculations. EHT5185 is an inactive analog of EHT1864. Plasma samples were spiked with EHT5185 as an internal standard, and extracted with ethyl acetate and dried under nitrogen. Dried samples were resuspended in acetonitrile for injection onto a Hypersil GOLD 50 mm x 2.1 mm, 1.9 µm column with 10 mm x 2.1 mm C18 guard cartridge. A gradient elution of 45-60% acetonitrile and 0.1% acetic acid with 55-40% 5 mM ammonium acetate and 0.3% acetic acid over 1 min was utilized at 0.5 mL/min on a Dionex Ultimate 3000 HPLC system. A TSQ Vantage tandem quadrupole mass spectrometer with a HESI-II probe, operated in multiple reaction monitoring mode, was used for positive ion detection and Xcalibur software was used for data acquisition and processing. MS/MS detection was conducted monitoring 509.160 → 212.020 m/z (collision energy 27 V) and 509.160 → 280.100 m/z (collision energy 23 V) for EHT-1864, and 508.171 → 241.950 m/z for EHT-5185 (collision energy 39 V). S-Lens RF amplitudes were 121 V and 132 V for EHT1864 and EHT5185, respectively. Source parameters were: spray voltage 500 V, vaporizer temperature 400°C, capillary temperature 280°C, sheath gas 30 and aux gas 4.

The calibration standards were linear over the range 0.0009-8.6 uM EHT1864, with quality controls (QCs) of 0.004, 0.043, 0.43, and 6.45 uM. Only calibration standards with >85% accuracy were used for quantitation. Intraday precision for low and high QCs were 14%CV and 2%CV respectively; intraday precision for low and high QCs were 17%CV and 6%CV respectively. Samples that exceeded the calibration range were diluted 50-fold

with blank plasma for reanalysis.The plasma EHT1864 concentration versus time data was initially inspected on log linear plot and attempted to be modeled to a multi-exponential decay model, but this could not be fitted due to sparse data. Thus non compartmental analysis was performed using WinNonLn, model 200. The pharmacokinetic parameters Tmax and Cmax were the observed time to mean maximum concentration, and maximum concentration, respectively and the elimination half -life was estimated using the whole data series

Genomics of drug sensitivity in cancer (GDSC) data mining

In the GDSC database (http://www.cancerrxgene.org/), 138 anti-cancer drugs were tested against up to 656 cancer cell lines *in vitro* [2]. Drug sensitivity data were downloaded. The profile of IC_{50} values across all cell lines in response to treatment with EHT1864 was compared to the individual IC_{50} profiles of each of the other 137 drugs using GraphPad Prism to generate Spearman correlation (*r*) values and *p*-values.

REFERENCES

1. Brachmann SM, Kleylein-Sohn J, Gaulis S, Kauffmann A, Blommers MJ, Kazic-Legueux M, Laborde L, Hattenberger M, Stauffer F, Vaxelaire J, Romanet V, Henry C, Murakami M, et al. Characterization of the mechanism of action of the pan class I PI3K inhibitor NVP-BKM120 across a broad range of concentrations. Molecular cancer therapeutics. 2012; 11: 1747-57.
2. Fiegl M, Samudio I, Mnjoyan Z, Korchin B, Fritsche H, Andreeff M. Physiological hypoxia promotes lipid raft and PI3K-dependent activation of MAPK 42/44 in leukemia cells. Leukemia. 2010; 24: 1364-7.
3. Garner F, Brown, J., Katzenellenbogen, J., Lyttle, C.R., Hattersley, G. Abstract P3-05-07: RAD1901, a novel oral, selective estrogen receptor degrader (SERD) with single agent efficacy in an ER+ primary patent derived ESR1 mutant xenograft model. Cancer Res. 2016; 76: Abstract nr P3-05-7.
4. Khaleel SS, Andrews EH, Ung M, DiRenzo J, Cheng C. E2F4 regulatory program predicts patient survival prognosis in breast cancer. Breast Cancer Res. 2014; 16.
5. Rothenfluh A, Cowan CW. Emerging roles of actin cytoskeleton regulating enzymes in drug addiction: actin or reactin'? Curr Opin Neurobiol. 2013; 23: 507-12.
6. Bokemeyer C, Bondarenko I, Makhson A, Hartmann JT, Aparicio J, de Braud F, Donea S, Ludwig H, Schuch G, Stroh C, Loos AH, Zubel A, Koralewski P. Fluorouracil, Leucovorin, and Oxaliplatin With and Without Cetuximab in the First-Line Treatment of Metastatic Colorectal Cancer. J Clin Oncol. 2009; 27: 663-71.

SUPPLEMENTARY FIGURES

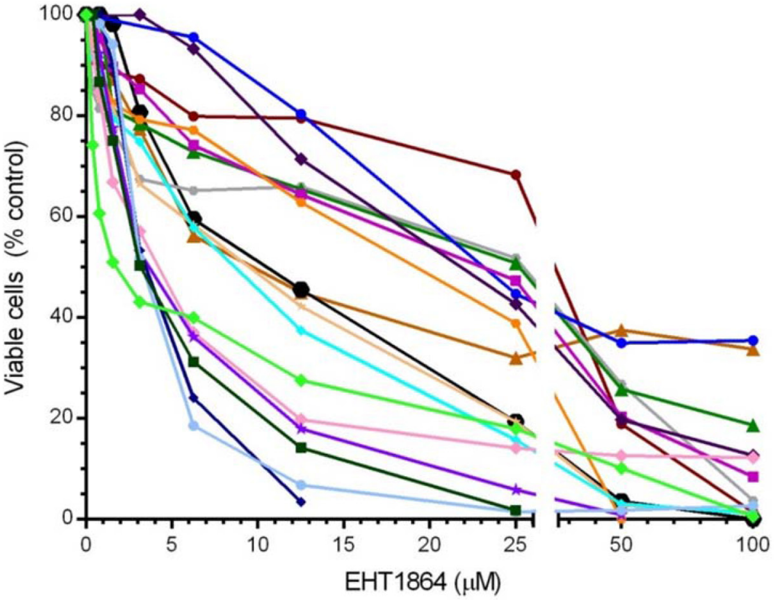

Cell Line	IC_{50} (μM)	Subtype	HER2	ER	*PIK3CA*	*PTEN*	*RAC1*	*RAC3*
ZR75-1	1.75	Lum	-	+	WT	L108R	WT	WT
MCF-7/FR	2.29	Lum	-	+	E545K	WT	WT	WT
BT-474	3.17	Lum	+	+	K111N	WT	WT	WT
HCC-1500	3.47	Lum	-	+	WT	WT	WT	amp
MCF-7	3.67	Lum	-	+	E545K	WT	WT	WT
T47D	4.23	Lum	-	+	H1047R	WT	WT	WT
BT-549	8.65	Basal (M)	-	-	WT	V275X	WT	WT
CAL-51	9.49	Basal (M)	-	-	E542K	N323fs E288fs	N39S	WT
MDA-MB-361	9.69	Lum	+	+	E545K	WT	WT	WT
SKBR3	10.5	HER2	+	-	WT	WT	WT	WT
MDA-MB-231	19.2	Basal (M)	-	-	WT	WT	WT	WT
MDA-MB-175-VII	21.8	Lum	-	+	WT	WT	WT	WT
HCC-1428	23.0	Lum	-	+	WT	WT	WT	WT
HCC-1395	23.1	Basal B	-	-	WT	N212fs	WT	amp
MDA-MB-415	25.7	Lum	-	+	WT	C136Y	WT	WT
CAMA-1	26.7	Lum	-	+	WT	D92H	WT	WT
CAL-120	34.2	Basal (M)	-	-	WT	WT	WT	amp

Supplementary Figure 1: Rac inhibition suppresses growth in *PIK3CA*-mutant and HER2+ breast cancer cells. Breast cancer cells were treated with 0-100 mM EHT1864 for 4-5 d. Relative viable cell numbers were assessed by SRB assay. Mutational and DNA copy number profiles were obtained from ref. [3]. The $RAC1^{N39S}$ mutation in CAL-51 cells is predicted to be low-impact per mutationassessor.org [4]. Subtype classifications were obtained from refs. [5, 6]. Lum- luminal; Basal (M)- basal mesenchymal-like; MCF-7/FR- fulv-resistant MCF-7 cells maintained and treated in 1 μM fulv.

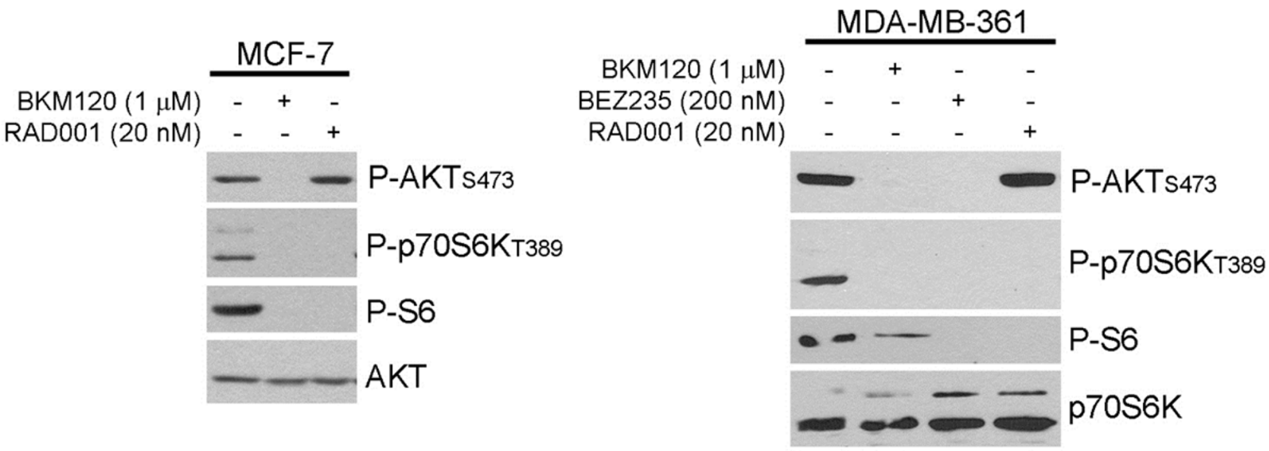

Supplementary Figure 2: PI3K/AKT signaling frequently drives mTORC1/p70S6K/S6 activation in *PIK3CA*-mutant and HER2+ breast cancer cells. Cells were treated with the PI3K inhibitor BKM120, the mTORC1 inhibitor RAD001, or the PI3K/mTOR dual inhibitor BEZ235 for 18-24 h. Lysates were analyzed by immunoblot.

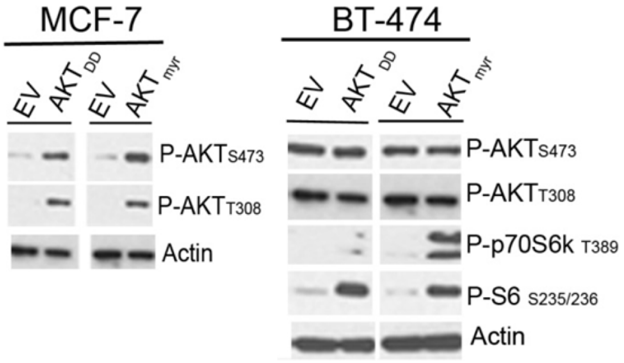

Supplementary Figure 3: Confirmation of recombinant protein expression in stably transfected cell lines. Cells were stably transfected with vectors encoding activated AKT1 (AKT_{DD} or AKT_{myr}), or empty vector control (EV). Lysates were analyzed by immunoblot.

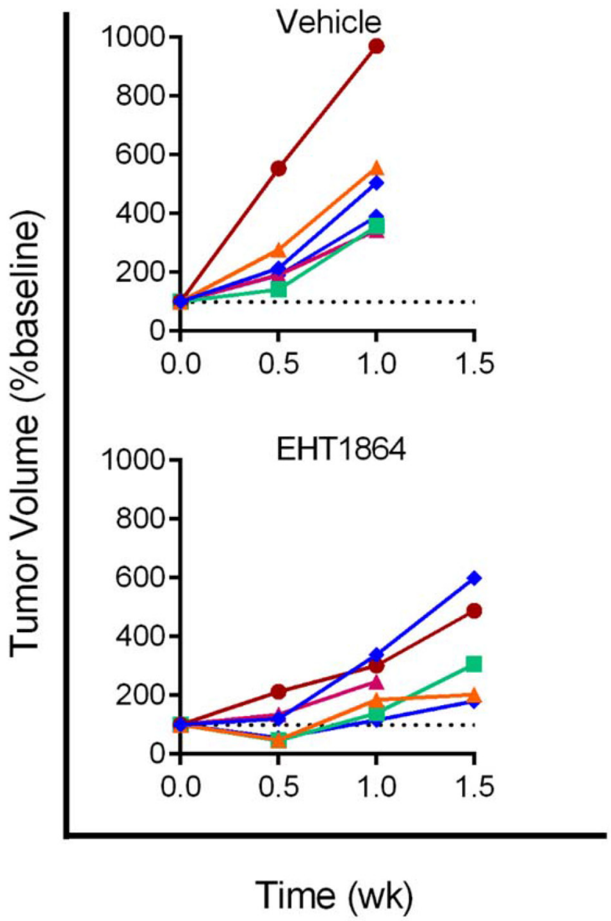

Supplementary Figure 4: Individual BT-474 tumor volumes. Mice bearing BT-474 tumors 200 mm^3 were randomized to drug treatments as indicated. Data are presented as % tumor volume relative to baseline. Each line represents an individual mouse.

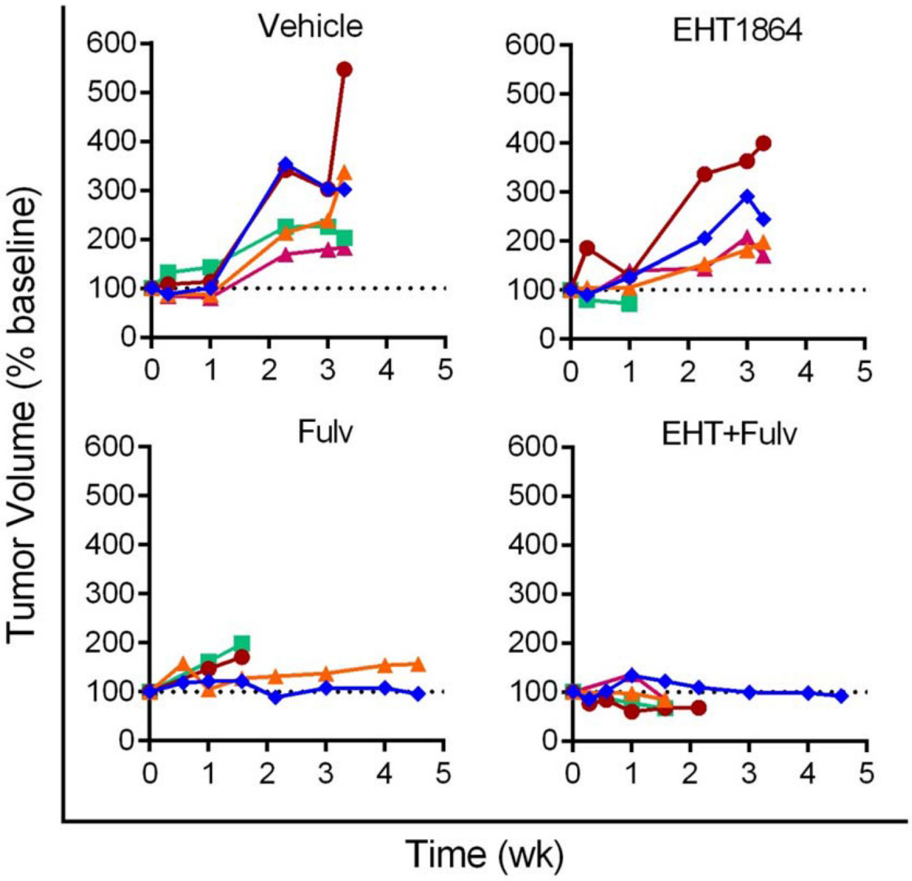

Supplementary Figure 5: Individual MCF-7 tumor volumes. Mice bearing MCF-7 tumors 200 mm^3 were randomized to drug treatments as indicated. Data are presented as % tumor volume relative to baseline. Each line represents an individual mouse.

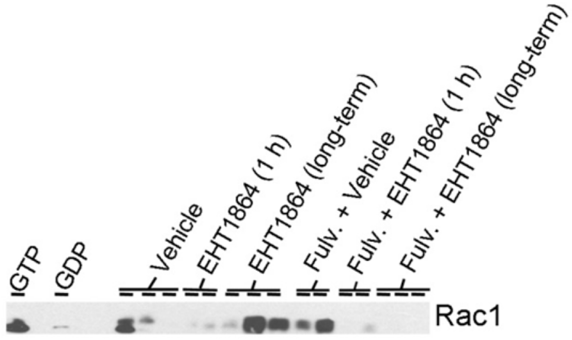

Supplementary Figure 6: EHT1864 inhibits Rac1 activation in tumors. Mice bearing MCF-7 tumors were treated +/− fulv for ~4 wk, and either treated with one dose of EHT1864 at 1 h prior to tumor harvest, or co-treated with EHT1864 for ~4 wk prior to tumor harvest. Tumors were harvested and snap frozen. Tumor lysates were used for Active Rac immunoprecipitation assay, followed by immunoblot of bead eluates for Rac1.
